# Chromatin Remodeling-Related PRDM1 Increases Stomach Cancer Proliferation and Is Counteracted by Bromodomain Inhibitor

**DOI:** 10.3390/jpm14030224

**Published:** 2024-02-20

**Authors:** Yu-Hsuan Hung, Hui-Ching Wang, Mei-Ren Pan, Li-Tzong Chen

**Affiliations:** 1 Department of Medical Research, Kaohsiung Medical University Hospital, Kaohsiung Medical University, Kaohsiung 807, Taiwan; 2 Division of Hematology and Oncology, Department of Internal Medicine, Kaohsiung Medical University Hospital, Kaohsiung Medical University, Kaohsiung 807, Taiwan; joellewang66@gmail.com; 3 Graduate Institute of Clinical Medicine, College of Medicine, Kaohsiung Medical University, Kaohsiung 807, Taiwan; mrpan@cc.kmu.edu.tw; 4 Drug Development and Value Creation Research Center, Kaohsiung Medical University, Kaohsiung 807, Taiwan; 5 National Institute of Cancer Research, National Health Research Institutes, Tainan 704, Taiwan; 6 Division of Gastroenterology, Department of Internal Medicine, Kaohsiung Medical University Hospital, Kaohsiung Medical University, Kaohsiung 807, Taiwan; 7 Center for Cancer Research, Kaohsiung Medical University, Kaohsiung 807, Taiwan

**Keywords:** PRDM1, stomach cancer, BET inhibitor, BRD4

## Abstract

Gastrointestinal (GI) cancers are some of the main public health threats to the world. Even though surgery, chemotherapy, and targeted therapy are available for their treatments, these approaches provide limited success in reducing mortality, making the identification of additional therapeutic targets mandatory. Chromatin remodeling in cancer has long been studied and related therapeutics are widely used, although less is known about factors with prognostic and therapeutic potential in such areas as gastrointestinal cancers. Through applying systematic bioinformatic analysis, we determined that out of 31 chromatin remodeling factors in six gastrointestinal cancers, only PR/SET domain 1 (PRDM1) showed both expression alteration and prognosis prediction. Analyses on pathways, therapies, and mediators showed that cell cycle, bromodomain inhibitor IBET151, and BET protein BRD4 were, respectively involved in PRDM1-high stomach cancer, while cell line experiments validated that PRDM1 knockdown in human stomach cancer cell line SNU-1 decreased its proliferation, BRD4 expression, and responsiveness to IBET151; accordingly, these results indicate the contribution by PRDM1 in stomach cancer formation and its association with BRD4 modulation as well as BET inhibitor treatment.

## 1. Introduction

Gastrointestinal (GI) cancers are some of the most menacing diseases globally [1,2], containing tumors that originate from the head and neck, stomach, liver, bile duct, pancreas, and colon [1,2]. In the US, cancers originating from the colon and pancreas rank 3rd and 4th in both sexes [3]; liver cancer ranks 5th in males and 7th in females; while head and neck cancers rank 7th in males. In Taiwan, cancers originating from the liver, colon, head and neck, pancreas, and stomach rank 2nd, 3rd, 6th, 7th, and 8th in terms of incidence, respectively. Common risk factors for GI cancers include obesity, smoking, and alcohol consumption [1,2] while unique risk factors also exist such as gastroesophageal reflux for stomach adenocarcinoma [4]. Additionally, GI cancers share similarities regarding genetic mutation and treatments [5,6,7] where mutations such as KRAS and TP53 are frequently observed among GI cancers [5]. Even though surgery [8], chemotherapy [9], radiotherapy [10], and targeted therapeutic [11] treatments are available, patients still confront issues of drug resistance and tumor metastasis [12,13], ensuring that novel therapeutic targets continue to warrant further investigation [6,7].

We focus on chromatin remodeling in GI cancer as it has been recently revisited as an important disease modulator and therapeutic target by us and other researchers [14,15]. For the storage of genetic information, chromatin is composed of the nucleosome, which contains DNA and histones [15,16], and for proper regulation of gene expression, nucleosomes are modulated by a chromatin remodeling complex in an ATP-dependent manner [16,17]. These chromatin remodeling complexes include switch/sucrose non-fermentable (SWI/SNF), initiator of SWI (ISWI), chromodomain helicase DNA binding protein (CHD), and INO80 [16], which are frequently mutated and participate in tumorigenesis in GI cancers.

Many therapeutics for epigenetic regulation such as inhibitors for bromodomains and extra terminal domain (BET) [18] and enhancers of zeste 2 polycomb-repressive complex 2 subunit (EZH2) [19], counteract chromatin remodeling-related GI cancers. To identify whether there are additional chromatin remodeling factors in GI cancers that might predict disease progression and offer treatment opportunities, we performed systematic bioinformatic analysis on The Cancer Genome Atlas (TCGA) datasets of GI cancers in attempting to determine along with our previous report [14] of chromatin remodeling gene list whether there was any potential target for disease prediction and treatment suggestion utilizing GEPIA for such analyses on target gene expression and prognosis prediction [20], thereby finding only PR/SET domain 1 (PRDM1) was upregulated in stomach cancer while predicting its poor prognosis.

PRDM1 is a chromatin remodeling-related transcription regulator [21,22] and was found to determine B cell fate in 2000 [21,22]. Afterward, PRDM1 was reported to affect blood cancers and was dysregulated in solid tumors [23,24,25,26,27,28,29,30,31,32,33]. In colon cancer, PRDM1 suppressed tumorigenesis via inhibition of TP53, MYC, and insulin-like growth factor-binding protein 3 (IGFBP3) [25,27,33], while in non-GI cancers, PRDM1 impeded the development of glioma [32], lung cancer [30], and melanoma [26]. Conversely, PRDM1 increased breast cancer invasiveness [29] and in GI cancer, PRDM1 ameliorated pancreatic tumor development via metastasis suppression [28]. These differences might be attributed to the diverse signals PRDM1 applied; for example, PRDM1 regulated the expressions of TP53 [33], CTNNB1 [32], CUL4A [31], MYC [27], and IGFBP3 [25], while on the other hand, revealing influence from HIF1A [28] and ERBB2 [29]. As the role of PRDM1 in GI cancers using systematic methodology has yet to be explored, we performed such bioinformatic analysis and found PRDM1 was increased in stomach cancer and this increment predicted poor prognosis; subsequently, we investigated the mechanism and therapeutic applications for PRDM1-high stomach cancer as well as briefly reviewing GI cancers and the potential roles of PRDM1 within.

For head and neck cancers, Johnson et al. reviewed the risk factors including tobacco, alcohol, environmental pollutants, and viral infection by the human papillomavirus or Epstein–Barr virus [34]. The Cancer Genome Atlas Network reported that typical oncogenes and tumor suppressors for this cancer type include cyclin-dependent kinase inhibitor 2A (CDKN2A), tumor protein p53 (TP53), phosphatidylinositol-4,5-bisphosphate 3-kinase catalytic subunit alpha (PIK3CA), notch receptor 1 (NOTCH1), and chromatin regulator lysine methyltransferase 2D (KMT2D) [35], while Shen et al. reported that PRDM1 is negatively associated with microsatellite instability in this cancer type [36].

For liver cancer, Llovet et al. reviewed the risk factors including viral infection by hepatitis virus (type B, C, or D), alcohol, nonalcoholic steatohepatitis, age, and gender [37], determining that liver cancer could also be classified into different molecular subclasses including progenitor, macro-trabecular massive, steato-hepatic, and cholestatic modalities. Gene mutation in the progenitor subclass is in axin 1 (AXIN1); for macro-trabecular massive subclass is in TSC complex subunit 1 or 2 (TSC1, TSC2) and the cholestatic subclass is in catenin beta 1 (CTNNB1). Mutations in the promoter of telomerase reverse-transcriptase (TERT) or gene body of TP53 span multiple subclasses [37]. Jia et al. reported that with previously identified 870 chromatin regulators, they developed a chromatin regulator-based prognostic risk score model that predicts survival status and associates with immunity as well as drug sensitivity in liver cancer [38]. 

Li et al. reported that PRDM1 induces cancer immune evasion via ubiquitin-specific peptidase 22 (USP22), Spi-1 proto-oncogene (SPI1), and programmed death ligand 1 (PDL1) while also continuing their previous works on PRDM1 resultantly finding it increased PDL1 expression in liver cancer. With tandem mass tag-based quantitative proteomic analysis, they found SPI1 was a modulator linking PRDM1 and PDL1, and as PRDM1 did not affect SPI1 mRNA expression, the authors explored its effect on protein expression and proteolysis, determining via transfection of ubiquitin-carrying plasmid that PRDM1 decreased SPI1 proteolysis. With the deubiquitinating enzyme (DUB) siRNA library, they found USP22 was the DUB that was affected by PRDM1 and then in turn affected SPI1 out of 98 DUBs. Ultimately, they applied single-cell RNA sequencing on liver cancer specimens and validated the relationship between PRDM1 expression and liver cancer immunity as well as treatment response [39].

For stomach cancer, Kumar et al. reviewed risk factors including age, *Helicobacter pylori* infection, tobacco use, and gender [40], while Slavin et al. reported that typical tumor suppressors for this cancer type included cadherin 1 (CDH1), serine/threonine kinase 11 (STK11), and SMAD family member 4 (SMAD4) [41]. Zeng et al. reported that DNA methylation is an important target in stomach cancer prediction and treatment while discovering that aberrantly methylated genes with potential for diagnosis and prognosis included secreted frizzle-related protein 2 (SFRP2), thrombospondin 1 (THBS1), ubiquitin C-terminal hydrolase L1 (UCHL1), SRY-box transcription factor 17 (SOX17), APC regulator of WNT signaling pathway (APC), E-cadherin, Ras association domain family member 1 (RASSF1A), ring finger protein 180 (RNF180), and spartin (SPART, SPG20) [42]. To our best knowledge, our present study is the first to report the effect of PRDM1 on stomach cancer formation.

For pancreatic cancer, Dr. Klein reviewed the risk factors including tobacco use, diabetes mellitus, obesity, alcohol consumption, pancreatitis, allergies, and familial history of cancer [43] while also determining that typical tumor suppressors for this cancer type include BRCA2 DNA repair-associated (BRCA2), BRCA1 DNA repair-associated (BRCA1), and CDKN2A [43]. Hayashi et al. further illustrated that lysine methyltransferase 2C (KMT2C), AT-rich interaction domain 1A (ARID1A), GATA binding protein 6 (GATA6), TP53, SMAD4, KRAS proto-oncogene, and GTPase (KRAS) are all involved in pancreatic cancer formation [44], while Kawakubo et al. revealed that the epigenetic regulation of pancreatic cancer could affect its response to immunotherapy, including the combined applications of inhibitors against DNMT, HDAC, BET, and EZH2 [45]. Chiou et al. applied a mouse model for pancreatic cancer and determined that in a highly metastatic subpopulation, PRDM1 is essential to maintain this phenotype. PRDM1 is regulated by hypoxia-inducible factor 1 subunit alpha (HIF1A), and the hypoxia-response element-containing region 240 kb upstream of the PRDM1 transcription initiation site is responsible for such regulation [28].

For colon cancer, Keum et al. reviewed the risk factors including obesity, dietary patterns, alcohol use, and tobacco use [46]. Li et al. determined that typical oncogenes and tumor suppressors for this cancer type included APC, KRAS, and TP53 [47], while Jung et al. discovered how DNA methylation and histone modifications and their inhibitors contributed to colon cancer formation and treatment [48]. For PRDM1 in colon cancer, Kim et al. reported that it promotes chemoresistance [25], Liu et al. reported that it inhibits proliferation [27], and Wan et al. reported that it induces cell cycle arrest [49].

With the above reports and reviews, the importance of GI cancers regarding development and treatment is well-documented, contributing to chromatin remodeling-related events in such circumstances. On the contrary, the role of PRDM1 in GI cancers has been less reported and our systematic bioinformatic analysis showed that its high expression in stomach cancer further predicted poor prognosis. This result inspired us to dissect the therapeutic and pathway modalities for PRDM1 in stomach cancer and validate them via wet-lab analysis.

## 2. Materials and Methods

### 2.1. GEPIA Analysis

To investigate the roles of chromatin remodeling-related factors in GI cancers, we applied such gene lists from our previous report [14] and analyzed their expression alterations and prognostic predictions in GEPIA [20]. Default settings for expressions as (1) |log_2_FC| > 1, (2) *p*-value < 0.01, and (3) log-scale as log_2_(TPM + 1) were applied along with those for prognosis as (1) overall survival and (2) median group cutoff. As PRDM1 in stomach cancer was the only chromatin remodeling-related factor displaying both expression alteration and prognosis prediction, its enriched pathways and therapeutics were further explored.

### 2.2. cBioPortal Analysis

The coexpressed genes for PRDM1-high stomach cancer in TCGA were extracted from cBioPortal [50], and the top five hundred positively or negatively correlated genes were uploaded to Reactome and L1000CDS^2^ for analyses on enriched pathways and therapeutics, respectively.

### 2.3. L1000CDS^2^ Analysis

The L1000CDS^2^ gene–drug interaction search engine [51] is based on the Connectivity Map and L1000 [52] to accelerate potential therapeutic identification. The above coexpression signature was uploaded and therapeutics having opposite signatures were identified for potential counteraction. As epigenetic inhibitors were enriched, especially those against BET, the most enriched BET inhibitor IBET151 was selected to test its effect on PRDM1-high stomach cancer.

### 2.4. Cell Culture and Reagents

Human stomach cancer cell line SNU-1 (60210) was obtained from the Biomaterial Collection Research Center (BCRC; Hsinchu, Taiwan). RPMI (SH30027.01) was obtained from Cytiva (Marlborough, MA, USA), fetal bovine serum (10437028), and penicillin-streptomycin-glutamine (10378016) from Gibco (Thermo Fisher Scientific; Waltham, MA, USA), trypan blue (T8154) from Sigma (Merck; Darmstadt, Germany), and bovine serum albumin (BSA; AD0023) from RAINBOW (Taipei, Taiwan).

### 2.5. RNA Interference

Cells were seeded in 6-well plates (92006, TPP; Zollstrasse, Switzerland) or 2-well chamber slides (154461, NUNC/Thermo Fisher Scientific), and transfected with 1 μg shRNA plasmid together with 3 μL HyFect (LDG0001RA, LEADGENE; Tainan, Taiwan). Two-day post-transfection cells were analyzed for target gene expression and proliferation. shRNAs were from RNA Technology Platform and Gene Manipulation Core Facility (RNAi core), Academia Sinica (Taipei, Taiwan) and the sequence for luciferase control was GCGGTTGCCAAGAGGTTCCAT, with the sequence for shPRDM1 being CATCTACTTCTACACCATTAA.

### 2.6. Cell Proliferation Assay

Cell proliferation was assayed by trypan blue exclusion as previously described [53]. A total of 200,000 SNU-1 cells were seeded in 6 wells and transfected with 1 μg indicated plasmid as well as 3 μL transfection reagent HyFect for two days. Transfection efficiency was confirmed by immunofluorescence and polymerase chain reaction (Supplementary Method). Cells were also subjected to trypan blue exclusion assay for proliferation analysis.

### 2.7. Immunofluorescence

As PRDM1 knockdown significantly decreased the proliferation of SNU-1 and rendered protein harvest unsuccessful even with triplicated cell number, we utilized immunofluorescence to analyze knockdown efficiency and BRD4 expression change. Two-day post-transfection cells were washed with PBS and fixed with 10% formalin for 10 min at room temperature, then, primary antibody was added at 1:100 dilution in 0.2% BSA-PBS wash buffer onto cells for overnight incubation at 4 °C. After washing with the buffer, cells were stained with fluorescent secondary antibody (GTX213110-04, GeneTex; Hsinchu, Taiwan) at 1:500 dilution for 1 h and DAPI (D9542, Sigma) at 5 μg/mL for 5 min at room temperature. Following washing with ddH_2_O, slides were mounted and the signals were analyzed using fluorescent microscopy (BX53, Olympus; Tokyo, Japan) and ImageJ (version 1.54f). Antibodies against PRDM1 (GTX132087) and BRD4 (GTX130586) were from GeneTex.

### 2.8. BET Inhibitor Treatment

IBET151 (HY-13235, MedChemExpress, Monmouth Junction, NJ, USA) at the lowest predicted concentration of 1 μM [54] was administrated onto cells and the effect of this inhibitor on PRDM1-high stomach cancer was assayed regarding proliferation.

### 2.9. Statistical Analysis

Statistical analyses were performed with GraphPad (Boston, MA, USA). The statistical difference between the control and experimental groups was analyzed with a *t*-test. *p* < 0.05 was considered statistically significant.

## 3. Results

### 3.1. PRDM1 Was Increased in Stomach Cancer and Predicted Poor Prognosis

To identify the roles of the chromatin remodeling-related factors in GI cancers in a systematic manner, GEPIA [20] was applied to assay expression alterations and prognosis predictions in TCGA datasets. These factors are mainly from families including DNA methyltransferase (DNMT), histone deacetylase (HDAC), PRDM, and protein arginine methyltransferase (PRMT). DNMTs control the methylation of DNA [55], and HDACs regulate the deacetylation of protein [56]. PRDMs manipulate gene transcription [57] while PRMTs control methylation on arginine [58]. Both histone and non-histone proteins are targets for HDACs [59] and PRMTs [60]. 

**Figure 1:**
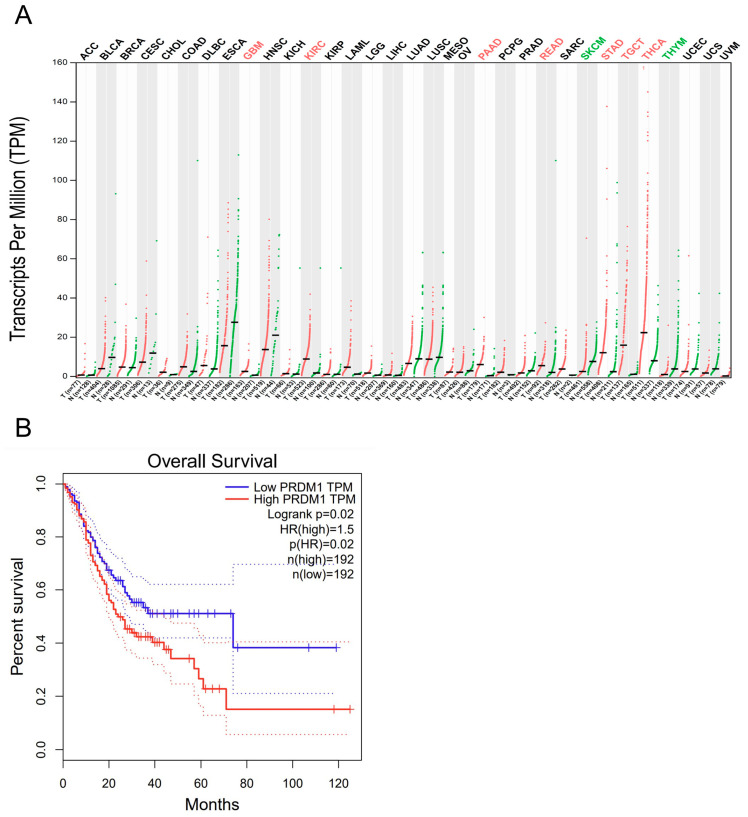
PRDM1 was increased in stomach cancer and predicted a poor prognosis. Chromatin remodeling-related factors identified from our previous report were analyzed with GEPIA for expression alterations and prognosis predictions across GI cancers in TCGA datasets. Among 31 candidates and six cancer types, only PRDM1 in stomach cancer showed both expression alteration (**A**) and prognosis prediction (**B**). In (**A**), those who fulfilled the criteria as |log_2_FC| > 1 and q value < 0.01 are emphasized with green color for downregulation and red color for upregulation.

**Figure A1:**
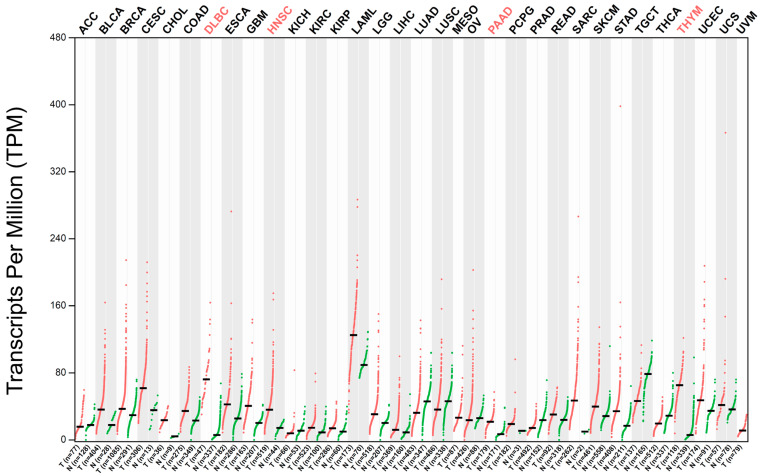
DNMT1 was increased in HNSC and PAAD. The expressions of DNMT1 from TCGA datasets were extracted from the GEPIA database.

**Figure A2:**
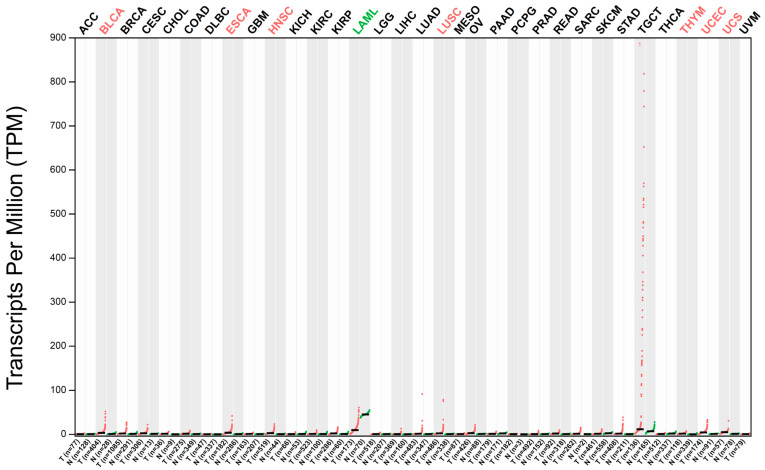
DNMT3B was increased in ESCA and HNSC. The expressions of DNMT3B from TCGA datasets were extracted from the GEPIA database.

**Figure A3:**
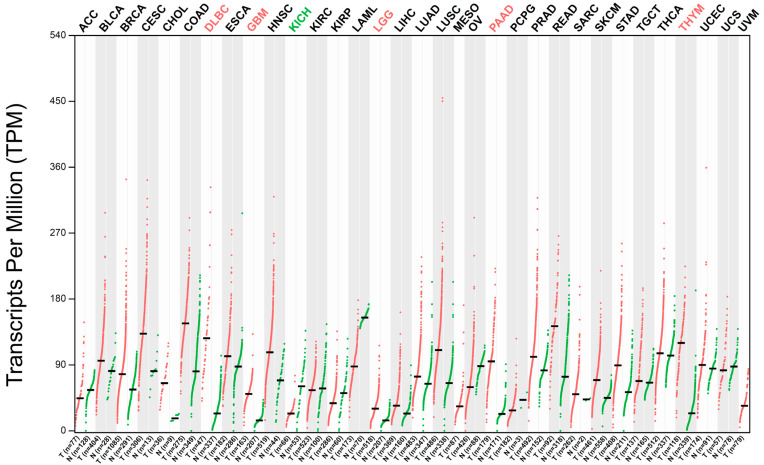
HDAC1 was increased in PAAD. The expressions of HDAC1 from TCGA datasets were extracted from the GEPIA database.

**Figure A4:**
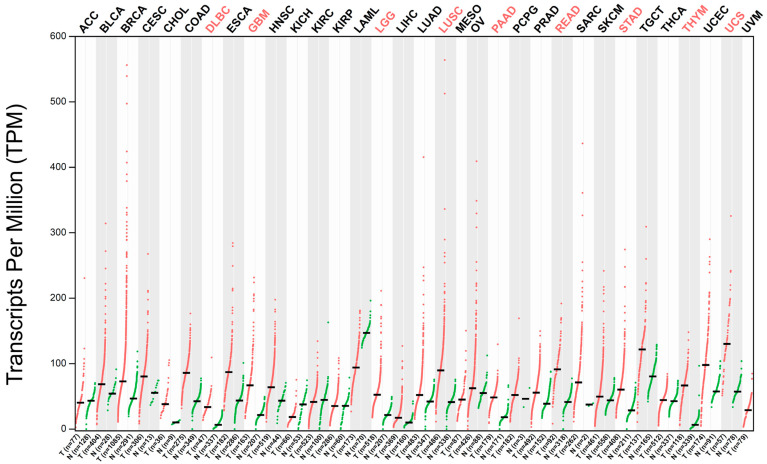
HDAC2 was increased in PAAD, READ, and STAD. The expressions of HDAC2 from TCGA datasets were extracted from the GEPIA database.

**Figure A5:**
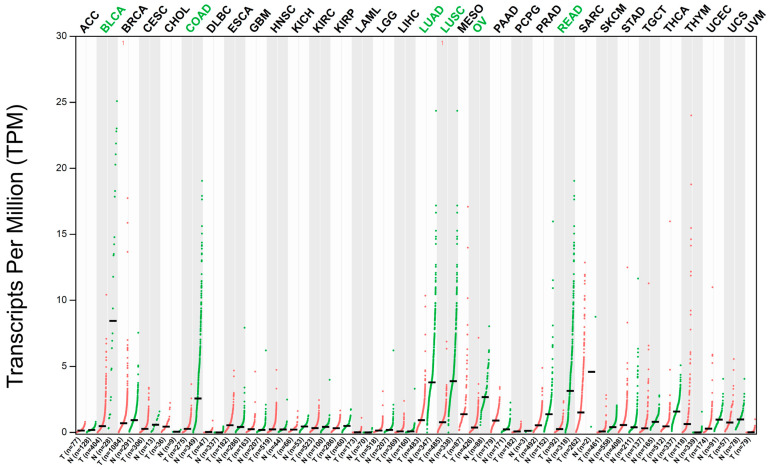
PRDM6 was decreased in COAD and READ. The expressions of PRDM6 from TCGA datasets were extracted from the GEPIA database.

**Figure A6:**
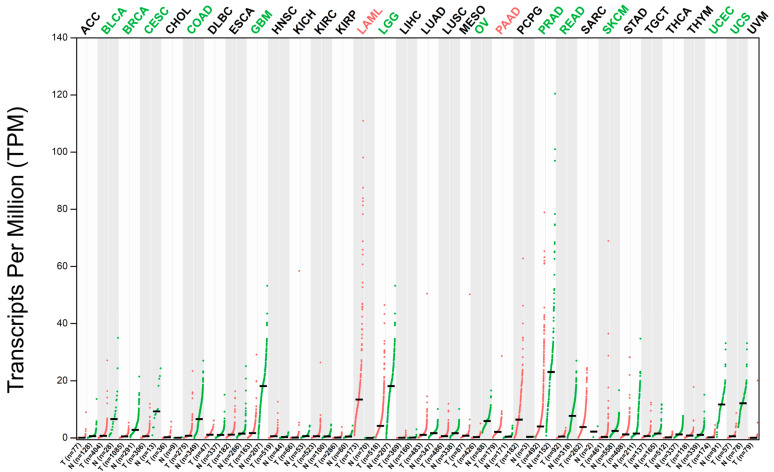
PRDM8 was altered in COAD, PAAD, and READ. The expressions of PRDM8 from TCGA datasets were extracted from the GEPIA database.

**Figure A7:**
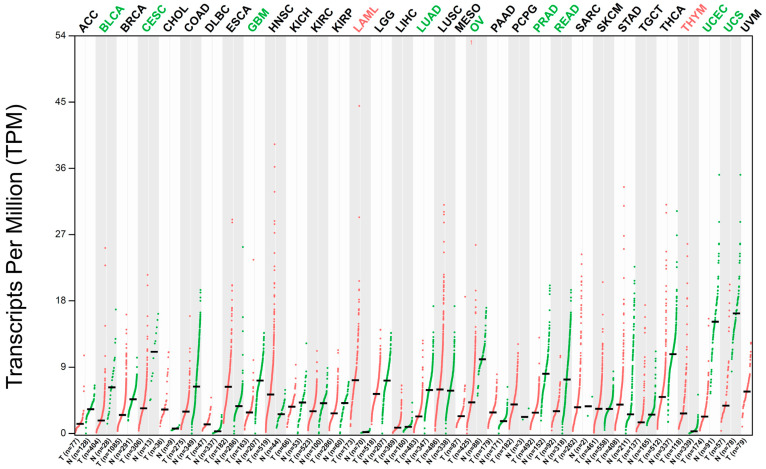
PRDM11 was decreased in READ. The expressions of PRDM11 from TCGA datasets were extracted from the GEPIA database.

**Figure A8:**
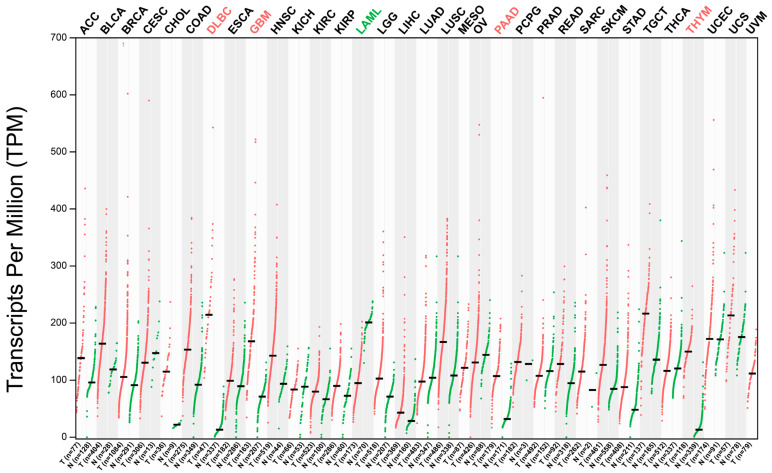
PRMT1 was increased in PAAD. The expressions of PRMT1 from TCGA datasets were extracted from the GEPIA database.

**Figure A9:**
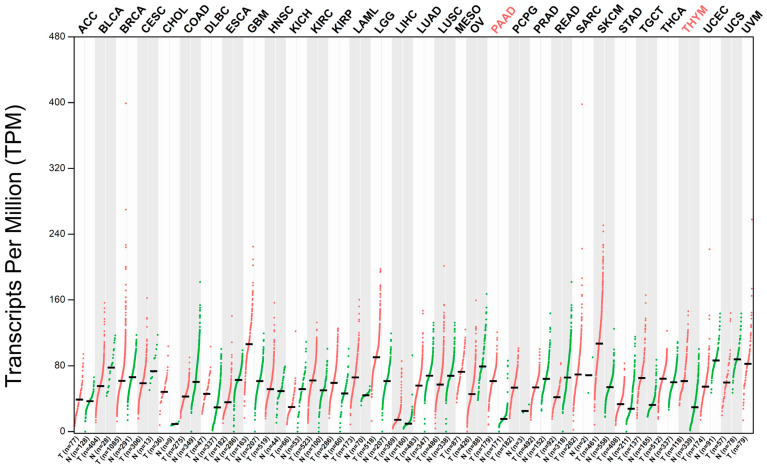
PRMT2 was increased in PAAD. The expressions of PRMT2 from TCGA datasets were extracted from the GEPIA database.

**Figure A10:**
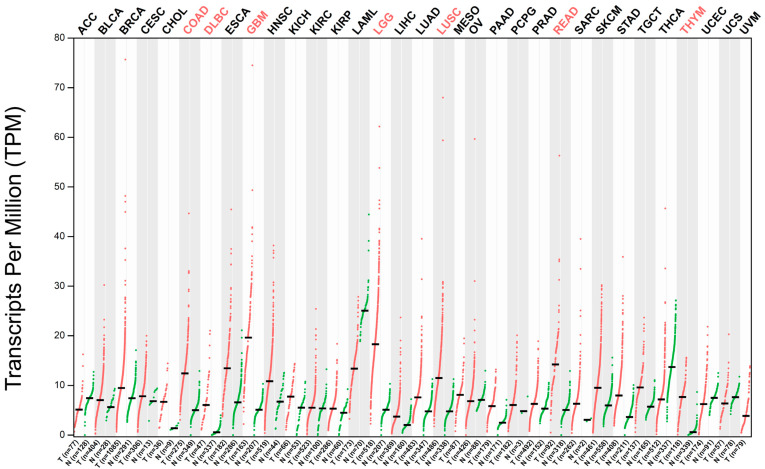
PRMT3 was increased in COAD and READ. The expressions of PRMT3 from TCGA datasets were extracted from the GEPIA database.

**Figure A11:**
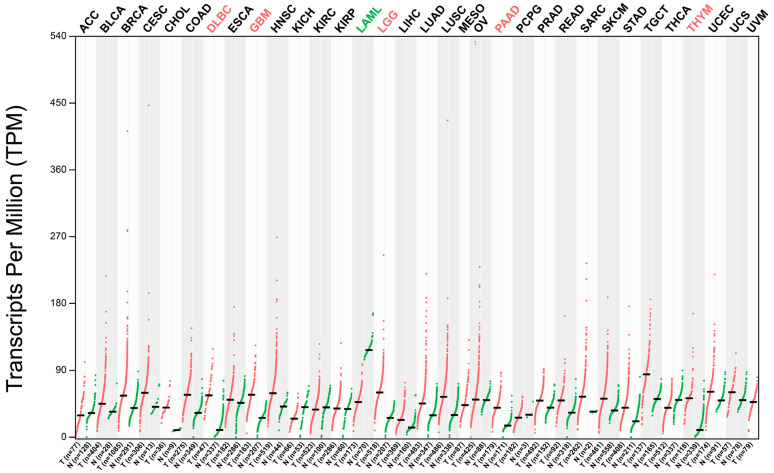
PRMT5 was increased in PAAD. The expressions of PRMT5 from TCGA datasets were extracted from the GEPIA database.

**Figure A12:**
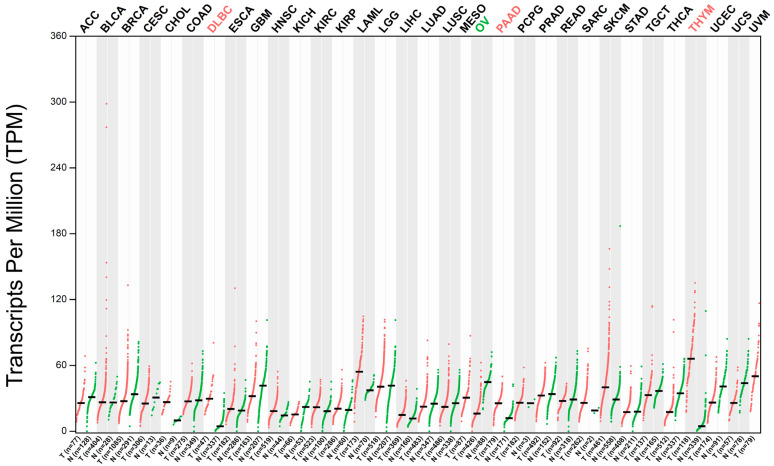
PRMT7 was increased in PAAD. The expressions of PRMT7 from TCGA datasets were extracted from the GEPIA database.

According to our previous report [14], the following factors are included in this analysis: DNMT1/2/3A/3B/3L, HDAC1/2/3, PRDM1/2/4/5/6/7/8/9/10/11/12/13/14/15/16, and PRMT1/2/3/5/6/7/8/10. As shown in Table 1, some of the chromatin remodeling-related factors were dysregulated during GI cancer formation. For DNMT, member 1 was upregulated in head and neck squamous cell carcinomas (HNSC) and pancreatic adenocarcinoma (PAAD), and member 3B was increased in HNSC and esophageal carcinomas (ESCA). In the HDAC family, member 1 was upregulated in PAAD, while member 2 showed increment additionally in rectum adenocarcinoma (READ) and stomach adenocarcinoma (STAD). For PRDMs, there were potential oncogenes and tumor suppressors in GI cancers, such as that member 1 was upregulated in PAAD, READ, and STAD while member 8 was increased in PAAD. On the contrary, PRDM8 as well as PRDM6 were downregulated in COAD and READ. READ is a cancer type with frequent PRDM dysregulations, as PRDM1 upregulation and PRDM6/8/11 downregulation were all observed. For PRMTs, members 1, 2, 5, and 7 were increased in PAAD, while member 3 was augmented in COAD and READ. Nevertheless, with all the expression alterations mentioned above, only PRDM1 in STAD predicted prognosis and was in accordance with its increment in this cancer type (Figure 1 and Figure A1, Figure A2, Figure A3, Figure A4, Figure A5, Figure A6, Figure A7, Figure A8, Figure A9, Figure A10, Figure A11 and Figure A12), so this result induced us to focus on PRDM1 in STAD as an important pair in chromatin remodeling-related factors in GI cancers.

### 3.2. PRDM1-High Stomach Cancer Was Enriched for Chromatin-Related Pathways and Was Targetable by BET Inhibitor In Silico

**Figure 2:**
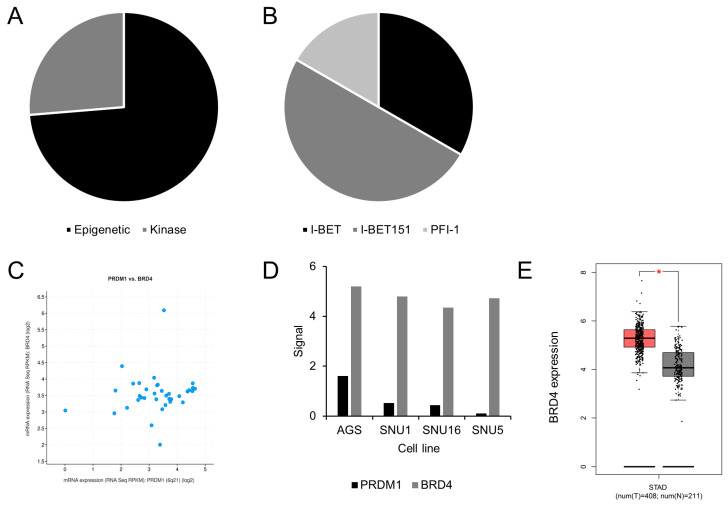
PRDM1-high stomach cancer was targeted by a BET inhibitor and was associated with BRD4 expression in silico. The therapeutic values for PRDM1-high stomach cancer were identified with the above co-expression signature and L1000CDS^2^. Identified therapeutics were divided according to their targets (**A**). In (**A**), the epigenetic therapeutics were BET inhibitors I-BET, I-BET151, and PFI-1 (**B**). The potential mediator for PRDM1 in stomach cancer was identified as BRD4 in cBioPortal (**C**) and CCLE (**D**), while the expression pattern of BRD4 in stomach cancer was identified with GEPIA (**E**). *, *p* < 0.01.

To further investigate the pathway and therapeutic strategies for PRDM1-high stomach cancer, we uploaded the above-mentioned co-expression signature to Reactome [61] and L1000CDS^2^, respectively. Reactome is a well-established pathway exploration database in cancer research [61], and L1000CDS^2^ [51] is based on a series of the Connectivity Map and L1000 [52] for signature-based therapeutic identification. We started with L1000CDS^2^ analysis in order to link back to the potential pathway once a candidate therapeutic was identified. While a few inhibitors possibly counteracting PRDM1-high stomach cancer were discovered, the most frequent ones were those for epigenetics (Figure 2A), with the targets of these epigenetic inhibitors all pointing to BET protein (Figure 2B).

**Figure 3:**
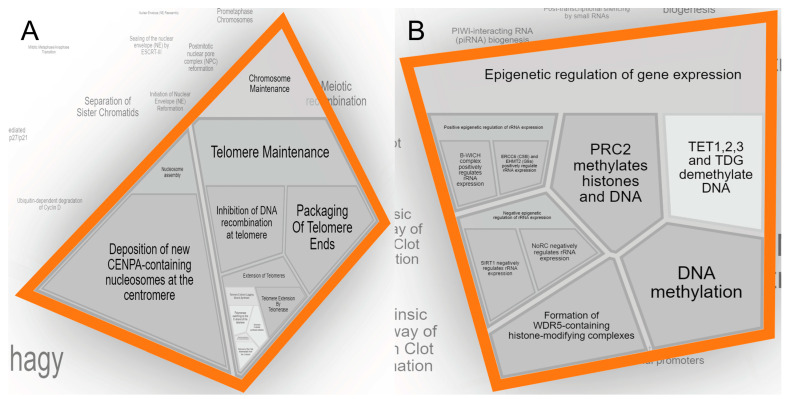
PRDM1-high stomach cancer was enriched for chromatin remodeling-related pathways. Reactome was applied to identify enriched pathways for PRDM1-high stomach cancer, and the results were overlapped with the therapeutic analysis mentioned in Figure 2 and shown as “cell cycle-chromosome maintenance” (**A**) and “gene expression (transcription)-epigenetic regulation of gene expression” (**B**).

These BET inhibitors that hampered the function of BET proteins such as BRD2, BRD3, and BRD4 [62] were initially identified in 2008. These proteins utilize their BET domains to regulate gene expression in development and disease [63], and their expressions are frequently dysregulated in the latter [64]. We wished to further investigate whether expressions of BET proteins were associated with that of PRDM1 in stomach cancer in the TCGA dataset and Cancer Cell Line Encyclopedia (CCLE), and such analysis with cBioPortal showed that BRD4 was positively associated with PRDM1 (Figure 2C), while BRD2 and BRD3 were not; additionally, PRDM1-high stomach cancer cell lines tended to express more BRD4 (Figure 2D). These cell lines [65,66] were selected due to their domestic availability so that the above observation could be validated. In the clinical aspect, BRD4 was also expressed at higher levels in the cancer portion rather than in the normal stomach (Figure 2E). Regarding pathway analysis, the presence of BET inhibitors as therapeutic for PRDM1-high stomach cancer allowed confirmation as to whether such pathways were enriched in the above coexpression signature; consequently, we uploaded this signature to Reactome [61] and found pathways including chromatin remodeling as well as epigenetic regulation were enriched in PRDM1-high stomach cancer (Figure 3). The figures were extracted from Reactome under the criteria of “Voronoi visualization” and “flattened view” [61,67]. As in Figure 3A and reference [68], WD repeat domain 5 (WDR5) is reported to participate in BRD4-regulated gene expression. The former factor affects histone trimethylation and the latter one is associated with histone acetylation [68]. The involvements of the chromatin remodeling-related pathway, BET inhibitor, and BRD4 expression in PRDM1-high stomach cancer galvanized us to test whether these associations could be validated in wet-lab assembly using SNU-1: the human stomach cancer cell line with high PRDM1 expression and level 1 biosafety.

### 3.3. PRDM1 Knockdown Decreased Cell Proliferation, BRD4 Expression, and IBET151 Sensitivity in Stomach Cancer

**Figure 4:**
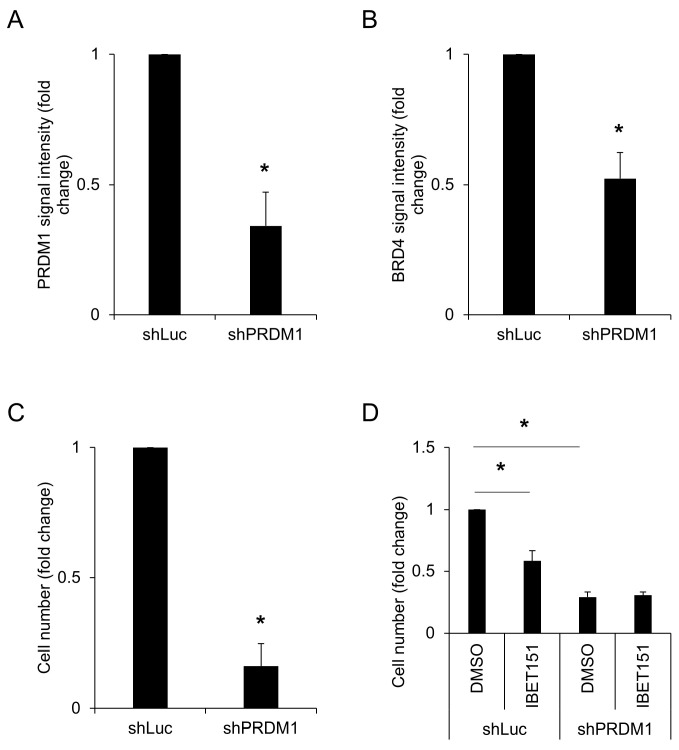
PRDM1 knockdown in stomach cancer decreased its BRD4 expression, proliferation, and response to BET inhibitor. Human stomach cancer cell line SNU-1 was transfected with shPRDM1 or shLuc control (**A**), and the effects on BRD4 expression (**B**), proliferation (**C**), and response to BET inhibitor (**D**) were analyzed two days later for post-transfection. *, *p*< 0.05.

Following bioinformatic analysis, we performed cell-line experiments to confirm the effects of PRDM1 on proliferation, BRD4 expression, and response to IBET151 in stomach cancer. Five shRNAs were screened and clone 71 was found to be effective in reducing PRDM1 expression in SNU-1 (Figure 4A and Figure A14). This shRNA was then utilized for further experimentation revealing that it greatly decreased expression of BRD4 in SNU-1 (Figure 4B), which was in accordance with the positive association between PRDM1 and BRD4 in TCGA and CCLE (Figure 3). shPRDM1 also decreased SNU-1 proliferation (Figure 4C), which echoed the association between PRDM1 expression and cell cycle progression in stomach cancer mentioned in Figure 2. We next tried whether RNA extraction would yield enough material to analyze the effect of shPRDM1 on the expressions of PRDM1 and BRD4, and indeed found RNA extraction was successful and shPRDM1 decreased the expression of not only itself but also that of BRD4 (Figure A14). When analyzing the band intensity of the product of polymerase chain reaction with ImageJ (version 1.54f) [69], the signals of PRDM1 and BRD4 were weaker in the group of shPRDM1. Whether BRD4 loss resulted from PRDM1 knockdown rendering stomach cancer insensitive toward IBET151 was of concern, so further testing found shPRDM1-SNU-1 displayed decreased sensitivity toward IBET151 while this inhibitor showed an obvious suppression on shLuc-SNU-1 in terms of proliferation (Figure 4D). PRDM1 expression thus indicated the potential effectiveness of BET inhibitors in stomach cancer treatment, as similar therapeutics have entered clinical trials [18].

## 4. Discussion

In the present study, we identified chromatin remodeling-related PRDM1 as a contributor to stomach cancer formation via modulations on cell proliferation and BRD4 expression; specifically, via systematic bioinformatic analysis on the chromatin remodeling-related gene list suggested by our team [14]. Besides, chromatin remodeling-related AT-rich interaction domain 1A (ARID1A) has been widely reported in stomach cancer formation. Lu et al. in 2023 mentioned that multiple ARID1A-related stomach cancer clinical trials were underway and that synthetic lethality combination for ARID1A-deficient stomach cancer might be inhibitors against PARP, PI3K, EZH2, and PD-L1 [70]. Yan et al. in 2014 found that ARID1A inhibited stomach cancer invasion by increasing β-catenin membrane translocation and E-cadherin transcription [71]. For other chromatin remodeling-related factors, Neil et al. in 2023 reported that SWI/SNF-related, matrix-associated, and actin-dependent regulator of chromatin, subfamily a, member 4 (SMARCA4) was mutated as truncated, unperceived, or mis-sensed in cancers of esophagus and stomach [72]. Liu et al. in 2023 reported that SWI/SNF-related, matrix-associated, and actin-dependent regulator of chromatin subfamily c member 1 (SMARCC1) was a poor prognosis predictor for overall and disease-free survival for stomach cancer and was further associated with invasion, lymph node involvement, and stage [73]. Hashimoto et al. in 2020 reported that chromodomain helicase DNA-binding protein 5 (CHD5) in stomach cancer was associated with pathological N status and good prognosis in both overall and recurrence-free survival [74] while also revealing that CHD5 overexpression decreased stomach cancer proliferation and invasion [74]. Our focus on PRDM1 in stomach cancer via systematic bioinformatic analysis and in vitro validation could add additional clues to chromatin remodeling factor-regulated stomach cancer appraisal.

For BET inhibitors in the treatment of gastrointestinal cancers, PRDM1 in stomach cancer is one of the potential targets, and Sun et al. in 2022 reviewed the effects of BET inhibitors on gastrointestinal cancers and their advancements in clinical trials [18], while Montenegro et al. in 2016 reported that an isoxazole PNZ5 might be a potential BET inhibitor and it increased stomach cancer apoptosis even in 3D spheroid [75].

To sum up, the present study utilized bioinformatic analysis and in vitro validation to show that PRDM1 increased stomach cancer formation by modulating cell proliferation and BRD4 expression but was counteracted by a BET inhibitor, which provides additional clues to stomach cancer research and treatment.

## 5. Conclusions

Summarizing the present study from systematic bioinformatic analysis for chromatin remodeling-related factors in GI cancers, we found PRDM1 in stomach cancer was the only pair showing expression alteration and prognosis prediction out of 31 candidate genes and six GI cancer types. Further therapeutic and pathway analyses revealed that PRDM1-high stomach cancer might be targeted by BET inhibitor and was enriched for chromatin remodeling-related features. Applying databases of cBioPortal and CCLE for proper in vitro validation, we found human stomach cancer cell lines tended to have greater BRD4 expressions once their PRDM1 expressions were higher, with this association also being observed in the TCGA STAD dataset. Cell-line experimentation indeed validated that PRDM1 knockdown in human stomach cancer cell line SNU-1 decreased BRD4 expression, proliferation, and sensitivity to BET inhibitor.

For basic research, we intend to perform promoter assay, chromatin immunoprecipitation, and RNA sequencing for shPRDM1-SNU-1. With promoter assay utilizing BRD4 promoter and shPRDM1-SNU-1, we can identify whether PRDM1 activates BRD4 promoter; with chromatin immunoprecipitation utilizing shPRDM1-SNU-1, we can identify whether PRDM1 binds to BRD4 promoter and whether this binding is lost after PRDM1 knockdown; and with RNA sequencing, we can identify how loss of PRDM1 in stomach cancer changes transcriptome, and with these clues, the source behind epigenetic regulation can be explored.

**Figure A13:**
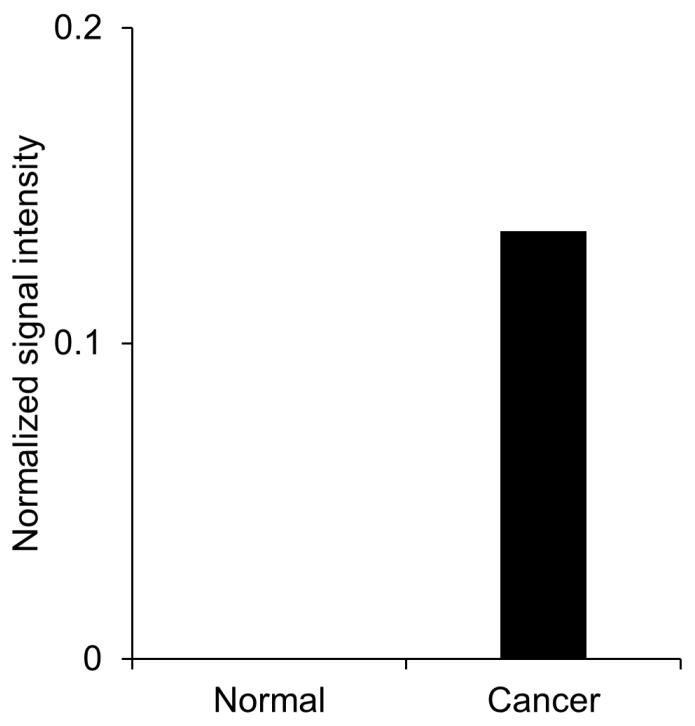
PRDM1 upregulation was observed in microarray GSE27342. For potential clinical application, we searched the NCBI gene expression omnibus database for systematic transcriptomic analysis on stomach cancer clinical specimens and found in GSE27342 Cui et al. analyzed 80 pairs of normal and cancerous stomach tissues, and we additionally found PRDM1 was upregulated in cancerous tissues in this dataset independent of TCGA STAD dataset.

**Figure A14:**
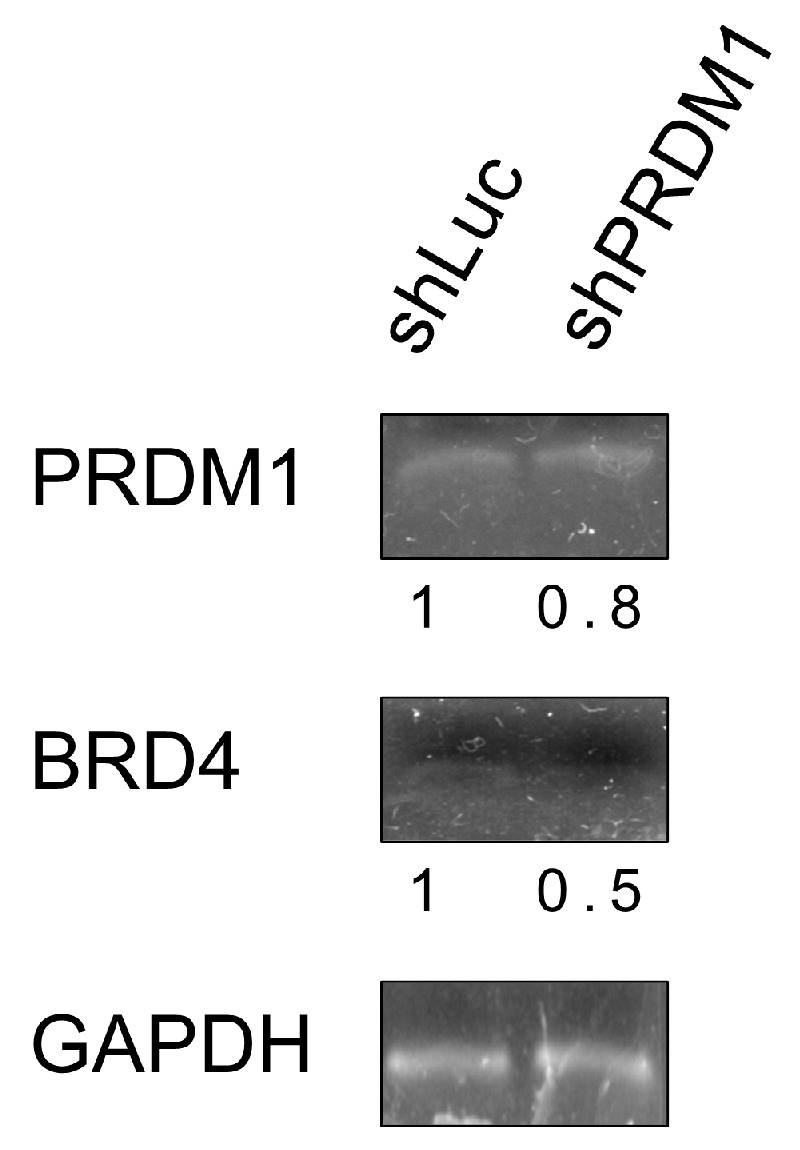
The effect of shPRDM1 on BRD4 expression at mRNA level. SNU-1 of shLuc or shPRDM1 clones was subjected to RNA extraction, and the effects of PRDM1 knockdown on expressions of PRDM1 and BRD4 were analyzed with polymerase chain reaction and gel electrophoresis. The signal intensities were analyzed and quantified with ImageJ (version 1.54f).

For clinical research, in the NCBI gene expression omnibus database [76], Cui et al. performed transcriptome analysis on 80 pairs of normal and cancerous stomach tissues with microarray [77], and from this result, we additionally identified that PRDM1 was upregulated in cancerous tissues as shown in Figure A13. This demonstrates that PRDM1 is detectable in stomach cancer in another independent cohort, and with up-to-date RNA sequencing on biopsy [78] or even liquid biopsy [79], the expression of PRDM1 could serve as a factor in the prediction of stomach cancer development and BET inhibitor treatment.

**Figure A15:**
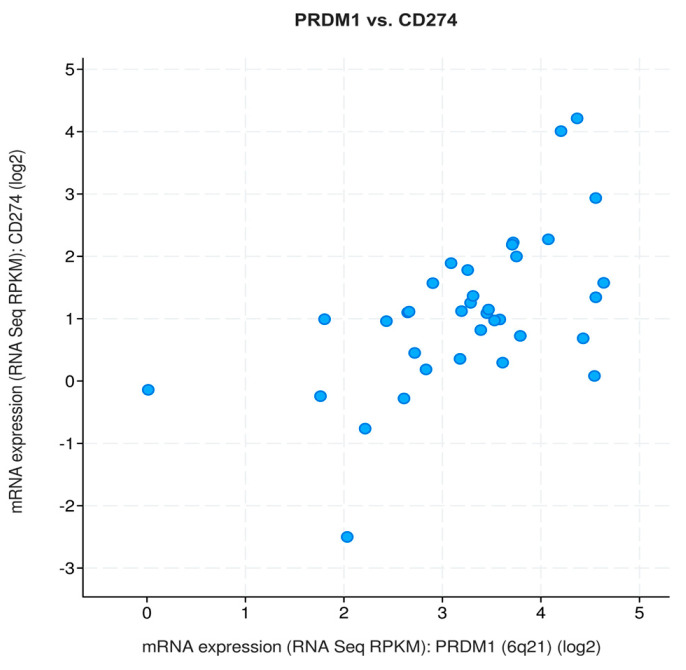
The positive correlation between PRDM1 and CD274 (PD-L1) in stomach cancer. The Spearman’s correlation, Pearson’s correlation, and their statistical significance for the relation between PRDM1 and CD274 were analyzed in the TCGA STAD dataset in cBioPortal database. Spearman correlation: 0.55 (*p* = 4.881 × 10^−4^). Pearson correlation: 0.57 (*p* = 2.993 × 10^−4^).

We additionally validated the positive correlation between PRDM1 and CD274 (programmed death ligand 1) in stomach cancer in the TCGA dataset (Figure A15). We also additionally reviewed the importance of chromatin remodeling in immunotherapy for GI cancers to further emphasize its clinical potential.

For head and neck cancer, Brennan et al. followed up their previous work and reported that nuclear receptor binding SET domain protein 1 (NSD1) was an indicator for the immunologically cold and DNA hypomethylated subtype of this cancer [80]. The inactivation of this transcriptional regulator led to decreased infiltration of T cells even in a mouse model, as the authors applied NOD-scid IL2Rgamma^null^ (NSG) mouse to establish tumor of control or NSD1 shRNA and human peripheral blood mononuclear cell (PBMC) injection.

For liver cancer, Shen et al. reviewed the potential enhancement by inhibitors against histone deacetylases on the efficiency of immunotherapy. These inhibitors included vorinostat and sodium valproate, which increased the expressions of PD-L1 and MHC class I polypeptide-related sequence B (MICB; for activation of natural killer cell), respectively [81]. On the contrary, Tao et al. reviewed how epigenetic regulation affected resistance to immunotherapy and addressed the molecular mechanisms within [82]. They emphasized that in liver cancer the inhibitors against DNA methyltransferase increased interferons and activated T cells, while the inhibitors against histone methyltransferase increased UL16 binding protein 1 (ULBP1) and activated natural killer cells. Chen et al. reported that chromatin organization-related gene signature predicted response to immunotherapy [83]. Wu et al. analyzed bioinformatically that there was a relation between genes for epigenetics and inflammation [84]. Cai et al. reported that the epigenetic regulator SWI/SNF-related, matrix-associated, and actin-dependent regulator of chromatin subfamily c member 1 (SMARCC1) was associated with decreased cytotoxic T cell and increased programmed cell death 1 (PDCD1, PD-1) [85].

For stomach cancer, Lin et al. analyzed bioinformatically the landscape of histone deacetylases and established the histone deacetylase score (HDS) model to predict immunotherapy response [86]. Yuan et al. reported that histone modification was involved in the stomach cancer subtype of stroma activation, which had a poor prognosis and immunotherapy resistance [87]. This epigenetic-modification-dysregulated (EMD) subtype employed mutations in chromatin regulators of the families of lysine methyltransferase, lysine demethylase, and histone deacetylase. Gu et al. reported that mutation in chromatin regulator AT-rich interaction domain 1A (ARID1A) was enriched for PD-1 signaling and showed a superior response to PD-1 blockade [88]. Wang et al. reported that mutation in lysine methyltransferase 2 was associated with PD-L1 positivity, cytotoxic lymphocyte, and efficiency of immune checkpoint inhibitor treatment [89].

For pancreatic cancer, Li et al. analyzed bioinformatically that lysine demethylase 5B (KDM5B) was increased in the tumor part and contributed to immunologically cold tumor microenvironment in respect of CD8^+^ T cell and interferon γ and validated with a subcutaneous mouse model [90]. Zhou et al. reported that histone deacetylase 5 (HDAC5) suppressed PD-L1 expression via deacetylation of lysine 310 residue of p65, with this deacetylation subsequently inhibited NF-κB activation as well as PD-L1 induction [91]. HDAC5 inhibition thus sensitized pancreatic cancer to PD-1 blockade [91].

## Figures and Tables

**Table 1:** Dysregulated chromatin remodeling-related factors in GI cancers.

Cancer/Gene	DNMT	HDAC	PRDM		PRMT
	Increment	Increment	Increment	Decrement	Increment
CHOL	-	-	-	-	-
COAD	-	-	-	6, 8	3
ESCA	3B	-	-	-	-
HNSC	1, 3B	-	-	-	-
LIHC	-	-	-	-	-
PAAD	1	1, 2	1, 8	-	1, 2, 5, 7
READ	-	2	1	6, 8, 11	3
STAD	-	2	1	-	-

CHOL, cholangiocarcinoma; COAD, colon adenocarcinoma; ESCA, esophageal carcinoma; HNSC, head and neck squamous cell carcinomas; LIHC, liver hepatocellular carcinoma; PAAD, pancreatic adenocarcinoma; READ, rectum adenocarcinoma; STAD, stomach adenocarcinoma.

## Data Availability

Data availability is listed as below: GEPIA (Gene Expression Profiling Interactive Analysis), http://gepia.cancer-pku.cn/ (accessed on 1 February 2024); cBioPortal for Cancer Genomics, https://www.cbioportal.org/ (accessed on 6 February 2024); L1000CDS^2^, https://maayanlab.cloud/L1000CDS2/#/index (accessed on 11 January 2024); Reactome Pathway Database, https://reactome.org/ (accessed on 1 February 2024).

## Supplementary Materials

The following supporting information can be downloaded at: https://www.mdpi.com/article/10.3390/jpm14030224/s1.

## References

[1] Huang J., Lucero-Prisno D.E., Zhang L., Xu W., Wong S.H., Ng S.C., Wong M.C. (2023). Updated epidemiology of gastrointestinal cancers in East Asia. Nat. Rev. Gastroenterol. Hepatol..

[2] Arnold M., Abnet C.C., Neale R.E., Vignat J., Giovannucci E.L., McGlynn K.A., Bray F. (2020). Global burden of 5 major types of gastrointestinal cancer. Gastroenterology.

[3] Siegel R.L., Miller K.D., Wagle N.S., Jemal A. (2023). Cancer statistics, 2023. CA Cancer J. Clin..

[4] Jardim S.R., de Souza L.M.P., de Souza H.S.P. (2023). The Rise of Gastrointestinal Cancers as a Global Phenomenon: Unhealthy Behavior or Progress?. Int. J. Environ. Res. Public Health.

[5] Wadhwa V., Patel N., Grover D., Ali F.S., Thosani N. (2023). Interventional gastroenterology in oncology. CA Cancer J. Clin..

[6] Smet A., Kupcinskas J., Link A., Hold G.L., Bornschein J. (2022). The role of microbiota in gastrointestinal cancer and cancer treatment: Chance or curse?. Cell Mol. Gastroenterol. Hepatol..

[7] Yang Z., Wang D., Zhang C., Liu H., Hao M., Kan S., Liu D., Liu W. (2022). The applications of gold nanoparticles in the diagnosis and treatment of gastrointestinal cancer. Front. Oncol..

[8] Bektaş M., Burchell G.L., Bonjer H.J., van der Peet D.L. (2023). Machine learning applications in upper gastrointestinal cancer surgery: A systematic review. Surg. Endosc..

[9] Hou J., Xie R., Zhang Z., Liu Q., Xiang Q., Cui Y. (2023). Hematologic side effects of immune checkpoint inhibitor with or without chemotherapy in patients with advanced and metastatic gastrointestinal cancer: A systematic review and network meta-analysis of phase 3 trials. Front. Pharmacol..

[10] Secerov Ermenc A., Segedin B. (2023). The Role of MRI and PET/CT in Radiotherapy Target Volume Determination in Gastrointestinal Cancers—Review of the Literature. Cancers.

[11] Pottier C., Fresnais M., Gilon M., Jérusalem G., Longuespée R., Sounni N.E. (2020). Tyrosine kinase inhibitors in cancer: Breakthrough and challenges of targeted therapy. Cancers.

[12] Chai C., Ji P., Xu H., Tang H., Wang Z., Zhang H., Zhou W. (2023). Targeting cancer drug resistance utilizing organoid technology. Biomed. Pharmacother..

[13] Cortes-Guiral D., Huebner M., Alyami M., Bhatt A., Ceelen W., Glehen O., Lordick F., Ramsay R., Sgarbura O., Van der Speeten K. (2021). Primary and metastatic peritoneal surface malignancies. Nat. Rev. Dis. Primers.

[14] Kuo C.Y., Moi S.H., Hou M.F., Luo C.W., Pan M.R. (2023). Chromatin Remodeling Enzyme Cluster Predicts Prognosis and Clinical Benefit of Therapeutic Strategy in Breast Cancer. Int. J. Mol. Sci..

[15] Li Z., Zhao B., Qin C., Wang Y., Li T., Wang W. (2022). Chromatin Dynamics in Digestive System Cancer: Commander and Regulator. Front. Oncol..

[16] Zhang F.L., Li D.Q. (2022). Targeting Chromatin-Remodeling Factors in Cancer Cells: Promising Molecules in Cancer Therapy. Int. J. Mol. Sci..

[17] Jancewicz I., Siedlecki J.A., Sarnowski T.J., Sarnowska E. (2019). BRM: The core ATPase subunit of SWI/SNF chromatin-remodelling complex-a tumour suppressor or tumour-promoting factor?. Epigenet. Chromatin.

[18] Sun H.-Y., Du S.-T., Li Y.-Y., Deng G.-T., Zeng F.-R. (2022). Bromodomain and extra-terminal inhibitors emerge as potential therapeutic avenues for gastrointestinal cancers. World J. Gastrointest. Oncol..

[19] Yu W., Liu N., Song X., Chen L., Wang M., Xiao G., Li T., Wang Z., Zhang Y. (2023). EZH2: An Accomplice of Gastric Cancer. Cancers.

[20] Tang Z., Li C., Kang B., Gao G., Li C., Zhang Z. (2017). GEPIA: A web server for cancer and normal gene expression profiling and interactive analyses. Nucleic Acids Res..

[21] Angelin-Duclos C., Cattoretti G., Lin K.I., Calame K. (2000). Commitment of B lymphocytes to a plasma cell fate is associated with Blimp-1 expression in vivo. J. Immunol..

[22] Yu J., Angelin-Duclos C., Greenwood J., Liao J., Calame K. (2000). Transcriptional repression by blimp-1 (PRDI-BF1) involves recruitment of histone deacetylase. Mol. Cell Biol..

[23] Santoro A., Bica M.G., Dagnino L., Agueli C., Salemi D., Cannella S., Veltroni M., Cetica V., Giarin E., Fabbiano F. (2009). Altered mRNA expression of PAX5 is a common event in acute lymphoblastic leukaemia. Br. J. Haematol..

[24] Zhu L., Kong Y., Zhang J., Claxton D.F., Ehmann W.C., Rybka W.B., Palmisiano N.D., Wang M., Jia B., Bayerl M. (2017). Blimp-1 impairs T cell function via upregulation of TIGIT and PD-1 in patients with acute myeloid leukemia. J. Hematol. Oncol..

[25] Kim J., Moon Y. (2021). Mucosal ribosomal stress-induced PRDM1 promotes chemoresistance via stemness regulation. Commun. Biol..

[26] Iwanaga R., Truong B.T., Hsu J.Y., Lambert K.A., Vyas R., Orlicky D., Shellman Y.G., Tan A.C., Ceol C., Artinger K.B. (2020). Loss of prdm1a accelerates melanoma onset and progression. Mol. Carcinog..

[27] Liu C., Banister C.E., Weige C.C., Altomare D., Richardson J.H., Contreras C.M., Buckhaults P.J. (2018). PRDM1 silences stem cell-related genes and inhibits proliferation of human colon tumor organoids. Proc. Natl. Acad. Sci. USA.

[28] Chiou S.H., Risca V.I., Wang G.X., Yang D., Grüner B.M., Kathiria A.S., Ma R.K., Vaka D., Chu P., Kozak M. (2017). BLIMP1 Induces Transient Metastatic Heterogeneity in Pancreatic Cancer. Cancer Discov..

[29] Sciortino M., Camacho-Leal M.D.P., Orso F., Grassi E., Costamagna A., Provero P., Tam W., Turco E., Defilippi P., Taverna D. (2017). Dysregulation of Blimp1 transcriptional repressor unleashes p130Cas/ErbB2 breast cancer invasion. Sci. Rep..

[30] Zhu Z., Wang H., Wei Y., Meng F., Liu Z., Zhang Z. (2017). Downregulation of PRDM1 promotes cellular invasion and lung cancer metastasis. Tumour Biol..

[31] Hung K.H., Su S.T., Chen C.Y., Hsu P.H., Huang S.Y., Wu W.J., Chen M.J., Chen H.Y., Wu P.C., Lin F.R. (2016). Aiolos collaborates with Blimp-1 to regulate the survival of multiple myeloma cells. Cell Death Differ..

[32] Wang X., Wang K., Han L., Zhang A., Shi Z., Zhang K., Zhang H., Yang S., Pu P., Shen C. (2013). PRDM1 is directly targeted by miR-30a-5p and modulates the Wnt/β-catenin pathway in a Dkk1-dependent manner during glioma growth. Cancer Lett..

[33] Yan J., Jiang J., Lim C.A., Wu Q., Ng H.H., Chin K.C. (2007). BLIMP1 regulates cell growth through repression of p53 transcription. Proc. Natl. Acad. Sci. USA.

[34] Johnson D.E., Burtness B., Leemans C.R., Lui V.W.Y., Bauman J.E., Grandis J.R. (2020). Head and neck squamous cell carcinoma. Nat. Rev. Dis. Primers.

[35] TCGA (2015). Comprehensive genomic characterization of head and neck squamous cell carcinomas. Nature.

[36] Shen L., Chen Q., Yang C., Wu Y., Yuan H., Chen S., Ou S., Jiang Y., Huang T., Ke L. (2020). Role of PRDM1 in Tumor Immunity and Drug Response: A Pan-Cancer Analysis. Front. Pharmacol..

[37] Llovet J.M., Kelley R.K., Villanueva A., Singal A.G., Pikarsky E., Roayaie S., Lencioni R., Koike K., Zucman-Rossi J., Finn R.S. (2021). Hepatocellular carcinoma. Nat. Rev. Dis. Primers.

[38] Jia X., Zhang G. (2022). Characterization of chromatin regulators in hepatocellular carcinoma to guide clinical therapy. Front. Genet..

[39] Li Q., Zhang L., You W., Xu J., Dai J., Hua D., Zhang R., Yao F., Zhou S., Huang W. (2022). PRDM1/BLIMP1 induces cancer immune evasion by modulating the USP22-SPI1-PD-L1 axis in hepatocellular carcinoma cells. Nat. Commun..

[40] Kumar S., Metz D.C., Ellenberg S., Kaplan D.E., Goldberg D.S. (2020). Risk Factors and Incidence of Gastric Cancer after Detection of Helicobacter pylori Infection: A Large Cohort Study. Gastroenterology.

[41] Slavin T.P., Weitzel J.N., Neuhausen S.L., Schrader K.A., Oliveira C., Karam R. (2019). Genetics of gastric cancer: What do we know about the genetic risks?. Transl. Gastroenterol. Hepatol..

[42] Zeng Y., Rong H., Xu J., Cao R., Li S., Gao Y., Cheng B., Zhou T. (2022). DNA Methylation: An Important Biomarker and Therapeutic Target for Gastric Cancer. Front. Genet..

[43] Klein A.P. (2021). Pancreatic cancer epidemiology: Understanding the role of lifestyle and inherited risk factors. Nat. Rev. Gastroenterol. Hepatol..

[44] Hayashi A., Hong J., Iacobuzio-Donahue C.A. (2021). The pancreatic cancer genome revisited. Nat. Rev. Gastroenterol. Hepatol..

[45] Kawakubo K., Castillo C.F., Liss A.S. (2022). Epigenetic regulation of pancreatic adenocarcinoma in the era of cancer immunotherapy. J. Gastroenterol..

[46] Keum N., Giovannucci E. (2019). Global burden of colorectal cancer: Emerging trends, risk factors and prevention strategies. Nat. Rev. Gastroenterol. Hepatol..

[47] Li J., Ma X., Chakravarti D., Shalapour S., DePinho R.A. (2021). Genetic and biological hallmarks of colorectal cancer. Genes Dev..

[48] Jung G., Hernández-Illán E., Moreira L., Balaguer F., Goel A. (2020). Epigenetics of colorectal cancer: Biomarker and therapeutic potential. Nat. Rev. Gastroenterol. Hepatol..

[49] Wan Z., Lu Y., Rui L., Yu X., Li Z. (2016). PRDM1 overexpression induce G0/G1 arrest in DF-1 cell line. Gene.

[50] de Bruijn I., Kundra R., Mastrogiacomo B., Tran T.N., Sikina L., Mazor T., Li X., Ochoa A., Zhao G., Lai B. (2023). Analysis and Visualization of Longitudinal Genomic and Clinical Data from the AACR Project GENIE Biopharma Collaborative in cBioPortal. Cancer Res..

[51] Duan Q., Reid S.P., Clark N.R., Wang Z., Fernandez N.F., Rouillard A.D., Readhead B., Tritsch S.R., Hodos R., Hafner M. (2016). L1000CDS(2): LINCS L1000 characteristic direction signatures search engine. NPJ Syst. Biol. Appl..

[52] Subramanian A., Narayan R., Corsello S.M., Peck D.D., Natoli T.E., Lu X., Gould J., Davis J.F., Tubelli A.A., Asiedu J.K. (2017). A Next Generation Connectivity Map: L1000 Platform and the First 1,000,000 Profiles. Cell.

[53] Hung Y.H., Hsu S.H., Hou Y.C., Chu P.Y., Su Y.Y., Shan Y.S., Hung W.C., Chen L.T. (2022). Semaphorin 6C Suppresses Proliferation of Pancreatic Cancer Cells via Inhibition of the AKT/GSK3/β-Catenin/Cyclin D1 Pathway. Int. J. Mol. Sci..

[54] Chaidos A., Caputo V., Gouvedenou K., Liu B., Marigo I., Chaudhry M.S., Rotolo A., Tough D.F., Smithers N.N., Bassil A.K. (2014). Potent antimyeloma activity of the novel bromodomain inhibitors I-BET151 and I-BET762. Blood.

[55] Del Castillo Falconi V.M., Torres-Arciga K., Matus-Ortega G., Díaz-Chávez J., Herrera L.A. (2022). DNA methyltransferases: From evolution to clinical applications. Int. J. Mol. Sci..

[56] Liu Y.M., Liou J.P. (2023). An updated patent review of histone deacetylase (HDAC) inhibitors in cancer (2020–present). Expert Opin. Ther. Pat..

[57] Di Tullio F., Schwarz M., Zorgati H., Mzoughi S., Guccione E. (2022). The duality of PRDM proteins: Epigenetic and structural perspectives. FEBS J..

[58] Dong J., Duan J., Hui Z., Garrido C., Deng Z., Xie T., Ye X.Y. (2022). An updated patent review of protein arginine N-methyltransferase inhibitors (2019–2022). Expert Opin. Ther. Pat..

[59] Shanmukha K.D., Paluvai H., Lomada S.K., Gokara M., Kalangi S.K. (2023). Histone deacetylase (HDACs) inhibitors: Clinical applications. Prog. Mol. Biol. Transl. Sci..

[60] Chen Q., Hu Q., Chen Y., Shen N., Zhang N., Li A., Li L., Li J. (2023). PRMT6 methylation of STAT3 regulates tumor metastasis in breast cancer. Cell Death Dis..

[61] Gillespie M., Jassal B., Stephan R., Milacic M., Rothfels K., Senff-Ribeiro A., Griss J., Sevilla C., Matthews L., Gong C. (2022). The reactome pathway knowledgebase 2022. Nucleic Acids Res..

[62] Bechter O., Schöffski P. (2020). Make your best BET: The emerging role of BET inhibitor treatment in malignant tumors. Pharmacol. Ther..

[63] Trojer P. (2022). Targeting BET bromodomains in cancer. Annu. Rev. Cancer Biol..

[64] Guo J., Zheng Q., Peng Y. (2023). BET proteins: Biological functions and therapeutic interventions. Pharmacol. Ther..

[65] Ji J., Chen X., Leung S.Y., Chi J.T.A., Chu K.M., Yuen S.T., Li R., Chan A.S., Li J., Dunphy N. (2002). Comprehensive analysis of the gene expression profiles in human gastric cancer cell lines. Oncogene.

[66] Park J.G., Frucht H., LaRocca R.V., Bliss Jr D.P., Kurita Y., Chen T.R., Henslee J.G., Trepel J.B., Jensen R.T., Johnson B.E. (1990). Characteristics of cell lines established from human gastric carcinoma. Cancer Res..

[67] Mohamed T.A., Elshamy A.I., Ibrahim M.A.A., Atia M.A.M., Ahmed R.F., Ali S.K., Mahdy K.A., Alshammari S.O., Al-Abd A.M., Moustafa M.F. (2021). Gastroprotection against Rat Ulcers by Nephthea Sterol Derivative. Biomolecules.

[68] Pistoni M., Rossi T., Donati B., Torricelli F., Polano M., Ciarrocchi A. (2021). Long Noncoding RNA NEAT1 Acts as a Molecular Switch for BRD4 Transcriptional Activity and Mediates Repression of BRD4/WDR5 Target Genes. Mol. Cancer Res..

[69] Schneider C.A., Rasband W.S., Eliceiri K.W. (2012). NIH Image to ImageJ: 25 years of image analysis. Nat. Methods.

[70] Lu S., Duan R., Cong L., Song Y. (2023). The effects of ARID1A mutation in gastric cancer and its significance for treatment. Cancer Cell Int..

[71] Yan H.B., Wang X.F., Zhang Q., Tang Z.Q., Jiang Y.H., Fan H.Z., Sun Y.H., Yang P.Y., Liu F. (2014). Reduced expression of the chromatin remodeling gene ARID1A enhances gastric cancer cell migration and invasion via downregulation of E-cadherin transcription. Carcinogenesis.

[72] Neil A.J., Zhao L., Isidro R.A., Srivastava A., Cleary J.M., Dong F. (2023). SMARCA4 Mutations in Carcinomas of the Esophagus, Esophagogastric Junction, and Stomach. Mod. Pathol..

[73] Liu S., Cao X., Wu S. (2022). High expression of SMARCC1 predicts poor prognosis in gastric cancer patients. Am. J. Cancer Res..

[74] Hashimoto T., Kurokawa Y., Wada N., Takahashi T., Miyazaki Y., Tanaka K., Makino T., Yamasaki M., Nakajima K., Mori M. (2020). Clinical significance of chromatin remodeling factor CHD5 expression in gastric cancer. Oncol. Lett..

[75] Montenegro R.C., Clark P.G., Howarth A., Wan X., Ceroni A., Siejka P., Nunez-Alonso G.A., Monteiro O., Rogers C., Gamble V. (2016). BET inhibition as a new strategy for the treatment of gastric cancer. Oncotarget.

[76] Barrett T., Wilhite S.E., Ledoux P., Evangelista C., Kim I.F., Tomashevsky M., Marshall K.A., Phillippy K.H., Sherman P.M., Holko M. (2013). NCBI GEO: Archive for functional genomics data sets—Update. Nucleic Acids Res..

[77] Cui J., Chen Y., Chou W.C., Sun L., Chen L., Suo J., Ni Z., Zhang M., Kong X., Hoffman L.L. (2011). An integrated transcriptomic and computational analysis for biomarker identification in gastric cancer. Nucleic Acids Res..

[78] Zhang P., Yang M., Zhang Y., Xiao S., Lai X., Tan A., Du S., Li S. (2020). Dissecting the Single-Cell Transcriptome Network Underlying Gastric Premalignant Lesions and Early Gastric Cancer. Cell Rep..

[79] Zhang Z., Wu H., Chong W., Shang L., Jing C., Li L. (2022). Liquid biopsy in gastric cancer: Predictive and prognostic biomarkers. Cell Death Dis..

[80] Brennan K., Shin J.H., Tay J.K., Prunello M., Gentles A.J., Sunwoo J.B., Gevaert O. (2017). NSD1 inactivation defines an immune cold, DNA hypomethylated subtype in squamous cell carcinoma. Sci. Rep..

[81] Shen C., Li M., Duan Y., Jiang X., Hou X., Xue F., Zhang Y., Luo Y. (2023). HDAC inhibitors enhance the anti-tumor effect of immunotherapies in hepatocellular carcinoma. Front. Immunol..

[82] Tao S., Liang S., Zeng T., Yin D. (2022). Epigenetic modification-related mechanisms of hepatocellular carcinoma resistance to immune checkpoint inhibition. Front. Immunol..

[83] Chen J., Chen X., Li T., Wang L., Lin G. (2022). Identification of chromatin organization-related gene signature for hepatocellular carcinoma prognosis and predicting immunotherapy response. Int. Immunopharmacol..

[84] Wu Z.H., Yang D.L., Wang L., Liu J. (2021). Epigenetic and Immune-Cell Infiltration Changes in the Tumor Microenvironment in Hepatocellular Carcinoma. Front. Immunol..

[85] Cai X., Zhou J., Deng J., Chen Z. (2021). Prognostic biomarker SMARCC1 and its association with immune infiltrates in hepatocellular carcinoma. Cancer Cell Int..

[86] Lin Y., Jing X., Chen Z., Pan X., Xu D., Yu X., Zhong F., Zhao L., Yang C., Wang B. (2023). Histone deacetylase-mediated tumor microenvironment characteristics and synergistic immunotherapy in gastric cancer. Theranostics.

[87] Yuan C., Zhang J., Deng C., Xia Y., Li B., Meng S., Jin X., Cheng L., Li H., Zhang C. (2022). Crosstalk of Histone and RNA Modifications Identified a Stromal-Activated Subtype with Poor Survival and Resistance to Immunotherapy in Gastric Cancer. Front. Pharmacol..

[88] Gu Y., Zhang P., Wang J., Lin C., Liu H., Li H., He H., Li R., Zhang H., Zhang W. (2023). Somatic ARID1A mutation stratifies patients with gastric cancer to PD-1 blockade and adjuvant chemotherapy. Cancer Immunol. Immunother..

[89] Wang J., Xiu J., Baca Y., Battaglin F., Arai H., Kawanishi N., Soni S., Zhang W., Millstein J., Salhia B. (2021). Large-scale analysis of KMT2 mutations defines a distinctive molecular subset with treatment implication in gastric cancer. Oncogene.

[90] Li X., Li J., Liu Y., Sun L., Tai Q., Gao S., Jiang W. (2024). Inhibition of KDM5B participates in immune microenvironment remodeling in pancreatic cancer by inducing STING expression. Cytokine.

[91] Zhou Y., Jin X., Yu H., Qin G., Pan P., Zhao J., Chen T., Liang X., Sun Y., Wang B. (2022). HDAC5 modulates PD-L1 expression and cancer immunity via p65 deacetylation in pancreatic cancer. Theranostics.

