# Supplementary material for: Chromatin Remodeling-Related PRDM1 Increases Stomach Cancer Proliferation and Is Counteracted by Bromodomain Inhibitor
Source: J Pers Med. 2024 Feb 20;14(3):224. doi: 10.3390/jpm14030224 (PMC10971448; doi:10.3390/jpm14030224)
Supplement: Supplementary file 1 [file jpm-14-00224-s001.zip › jpm-2843508-supplmentary.pdf]

Journal of
Personalized Medicine

Supplementary Materials

RNA Extraction, Reverse Transcription, and Polymerase Chain Reaction

RNA from cells of indicated conditions was extracted with Blood/Cell Total RNA Mini Kit (RB050) according to the manufacturer's instructions (Geneaid; New Taipei City, Taiwan). Reverse transcription with 2 μg RNA was performed with ReverTra Ace set (PU-TRT-100) according to the manufacturer's instructions (PURIGO; Taipei, Taiwan). Polymerase chain reaction was performed with Vazyme (Nanjing, China) 2 × Taq Master Mix (P111) under the condition of 94 °C, 1 min; 60 °C, 1 min; 72 °C, 1 min for 35 cycles. The result was analyzed with 1% agarose gel and ChemiDoc XRS+ (Bio-Rad; Hercules, CA, USA). Primer sequences were listed as below: PRDM1 -F, AAGCAACTGGATGCGCTATGT; PRDM1 -R, GGGATGGGCTTAATGGTGTAGAA; BRD4 -F, TGGATGCCGTCAAGCTGAAC; BRD4 -R, GTTTCTTCTGTGGGTAGCTCATT; GAPDH -F, AGAAGGCTGGGGCTCATTTG; GAPDH -R, AGGGGCCATCCACAGTCTTC.
